# Effect of a prolonged slow expiration technique on 24-h food intake in children hospitalized for moderate bronchiolitis: a randomized controlled trial

**DOI:** 10.1186/s13052-024-01770-2

**Published:** 2024-09-27

**Authors:** Yann Combret, Margaux Machefert, Mélody Couet, Tristan Bonnevie, Francis-Edouard Gravier, Timothée Gillot, Pascal Le Roux, Roger Hilfiker, Clément Medrinal, Guillaume Prieur

**Affiliations:** 1 Physiotherapy Department, Le Havre Hospital, Le Havre, F-76600 France; 2 ERPHAN, Paris-Saclay University, UVSQ, Versailles, F-78000 France; 3 School of Physiotherapy, Rouen University Hospital, Rouen, F-76000 France; 4 Univ Rouen Normandie, Normandie Univ, GRHVN UR 3830, Rouen, F-76000 France; 5 ADIR Association, Rouen University Hospital, Rouen, F-76000 France; 6 Normandie Univ, UNIROUEN, CETAPS EA3832, F-76000 Rouen, France; 7 Paediatric Department, Le Havre Hospital, Le Havre, F-76600 France; 8 Research and Independent Studies in Private Physiotherapy (RISE), 3902 Brig, Switzerland

**Keywords:** Chest physiotherapy, Airway clearance techniques, Feeding issues, Acute viral bronchiolitis

## Abstract

**Background:**

Chest physiotherapy for airway clearance is not recommended in children hospitalized with bronchiolitis. The updated Cochrane meta-analysis suggests that slow expiratory techniques could slightly improve clinical severity, but the evidence certainty is low and the clinical significance of this change is unknown. We investigated whether the prolonged slow expiration technique (PSET) would impact the 24-h food intake of these children.

**Methods:**

We conducted a two-arm double-blind randomized controlled trial. Hospitalized children aged from 1 to 12 months, bottle-fed or diversified and referred for airway clearance were included. Both groups received upper airway clearance at inclusion and standard treatments. The experimental group received PSET including rhinopharyngeal unclogging and targeted unprovoked cough. The primary outcome was the 24-h food intake. Clinical severity, vomit episodes and sleep quality were also recorded. An ordinary least squares linear regression for quantitative variables was modelled for between-group comparisons.

**Results:**

From January 9, 2019, to December 1, 2022, 42 children were randomized with a 1:1 ratio (mean age: 5.0 (± 2.9) months). The 24-h food intake did not differ between groups (estimate: 1.8% (95%CI -7.0 to 10.6); *p* = 0.68). PSET had no effect on SpO2, clinical severity, RR and HR at the follow-up assessments (5 min, 30 min and 24 h after intervention), nor on the number of vomit episodes, total sleep time and SpO2 during sleep.

**Conclusions:**

PSET did not affect food intake or the 24-h course of bronchiolitis more than standard treatment in children hospitalized for moderate bronchiolitis.

**Trial registration:**

NCT03738501 registered on 13/11/2018, Slow Expiratory Technique to Improve Alimentation in Children With Bronchiolitis (BRONCHIOL-EAT); https://classic.clinicaltrials.gov/ct2/show/NCT03738501.

**Supplementary Information:**

The online version contains supplementary material available at 10.1186/s13052-024-01770-2.

## Background

Acute viral bronchiolitis (AVB) is the most common respiratory disease in infants [1]. AVB usually begins with symptoms of a viral upper respiratory infection, such as nasal obstruction and rhinorrhea. This initial phase is followed by progressive bronchiolar obstruction caused by inflammation, oedema, mucus plugging and bronchospasm, leading to lower respiratory tract symptoms of persistent cough, increased work of breathing, impaired feeding and sleeping [2, 3]. AVB is self-limiting in the majority of cases, and only a minority of infants require hospitalisation [4]. Several medical treatments have shown no efficacy on symptoms, making supportive care (nutrition/hydration and oxygenation) and upper airway clearance the current recommended treatments worldwide [5, 6].

Among other non-pharmacological treatments, airway clearance techniques (ACT) have long been prescribed for children with AVB [7, 8]. ACT could assist children in the clearance of tracheobronchial secretions in order to decrease airway obstruction and improve respiratory mechanics. The 2023 updated Cochrane meta-analysis contradict this hypothesis and stated that ACT have no impact on hospital length of stay or time to recovery [9]. Unsurprisingly, in line with these conclusions, ACT is no longer recommended in many countries worldwide, except for children with comorbidities [5, 6, 10, 11]. On the other hand, new and gentler passive slow expiratory techniques have gained interest in the last decade. The Cochrane review reported that these techniques (i.e., the prolonged slow expiration technique (PSET) including retrograde rhinopharyngeal unclogging or not, and the autogenic assisted drainage (AAD)) may transiently and slightly decrease clinical severity [9, 12–14]. However, the magnitude of this change is low, the respiratory scores are heterogenous among studies and the level of evidence is low. To assess the clinical relevance of this change in clinical severity, we hypothesized that a relevant improvement should be reflected in higher food ingestion and better sleeping.

Therefore, our primary aim was to assess the effect of PSET including retrograde rhinopharyngeal unclogging on the 24-h food intake of infants hospitalized with moderate AVB. We also investigated the impact of PSET on the clinical severity, the respiratory rate (RR), the heart rate (HR), the pulsed oxygen saturation (SpO2), the number of vomit episodes and the sleep quality.

## Methods

### Study design

**Fig. 1:**
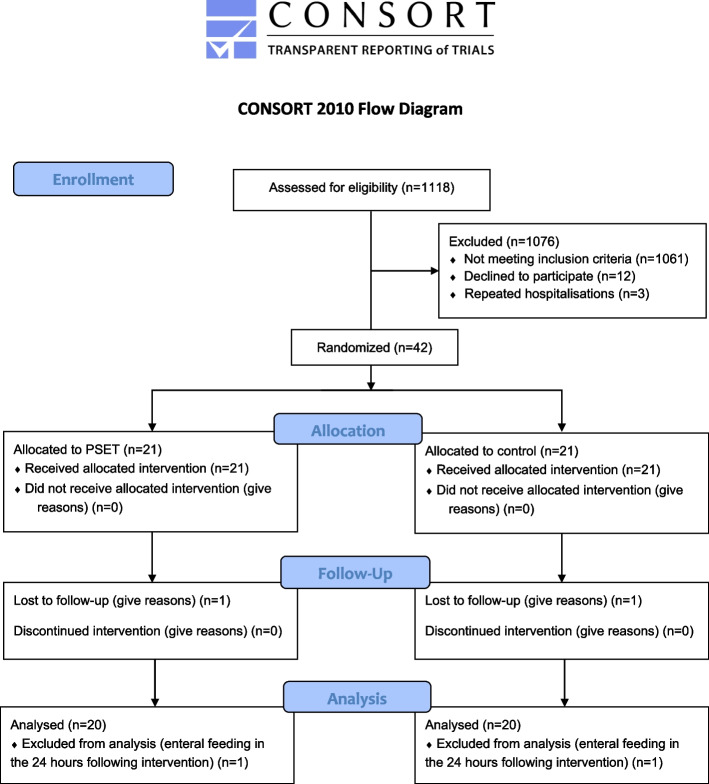
CONSORT flow diagram of participants

We conducted a single centre, parallel-group, double-blind randomized controlled trial with a 1:1 ratio in Le Havre Hospital (France), from January 2019 to December 2022. Ethical approval was granted by the French Comité de Protection des Personnes Sud-Méditerranée V (18.064), and written informed consent was obtained from one of the parents. The study was prospectively registered (NCT03738501) and is reported according to the CONSORT guidelines [15]. The CONSORT flow diagram of the study is presented in the Fig. 1 and the CONSORT checklist is presented in the additional file 3.

### Patient and public involvement

Parents’ experiences were involved in the choice of the primary outcome for this study. Feeding difficulties is stressful for parents of children with AVB, resulting in an important mental burden. Parents were then blinded to the intervention, but they were asked to report the 24-h feeding fraction of their child, as well as the number of vomits during follow-up. Finally, parents were advised that they could be informed of the final results of the study by the corresponding author.

### Eligibility and enrolment

We included all children who fulfilled the following criteria at the eligibility assessment: 1) aged 1–12 months, 2) hospitalized for AVB, 3) moderate clinical severity (respiratory score [16] ranging from 4 to 6), 4) < 24 h in hospital with an expected LOS > 24 h, 5) bottle-fed or diversified, and 6) referred for ACT by a physician not involved in the study. An experienced senior physiotherapist (YC) confirmed the potential benefit of ACT before inclusion based on stethoscope examination, subjective cough assessment and respiratory sounds. Non-inclusion criteria were: mixed or exclusive breastfeeding, gestational age < 35 weeks, cardiac, neurological or pulmonary comorbidities, oxygen supplementation or enteral feeding and contraindications to ACT. Children discharged from hospital, who received oxygen or nutritional supplementation during the follow-up, or who experienced side effects during the ACT (HR < 100 bpm or SpO2 < 90%) were excluded.

### Interventions

The interventions were all performed by a physiotherapist with > 5 years’ experience in ACT (YC), at least 2 h after the last feed. At inclusion, each group underwent upper airway clearance with a nasal instillation of 5 mL of isotonic solution delivered at low pressure through a syringe in side lying. A second 5 mL was added if the liquid coming out of the nostrils was not visually clear (total volume of 10 mL maximum per nostril), and this entire procedure was repeated in each nostril [17]. A retrograde rhinopharyngeal clearance (RRC) manoeuvre was added at the end of each instillation. At the end of exhalation, the physiotherapist raised the child’s lower jaw with his hand to briefly close the mouth, utilizing the inspiratory reflex to induce nasal aspiration [18].

The children in the PSET group then underwent a 10-min session of prolonged slow expiration technique. PSET was applied with one hand on the chest and the other hand below the umbilicus, according to previously described procedures [14]. A gentle, gradually increasing pressure was applied during expiration using both hands to mobilize the expiratory reserve volume for 2–3 breathing cycles. Rests of 5–10 spontaneous breaths were allowed between each PSET set. As it was previously described by Conesa-Segura et al., the technique involved retrograde rhinopharyngeal unclogging using a forced inspiratory maneuver designed to clear the nasopharynx [14]. Coughing was expected at the end of the manoeuvres, or triggered by changing body position, but not manually provoked to avoid any side effects related to this procedure [19]. The children in the control group were kept seated or lying calmly for 10 min in their room to preserve blinding.

Standard treatments were provided to both groups: nasal instillation applied by parents or nurses before feeding, medical treatments prescribed by a physician who was not involved in the study, and parental education about upper airway clearance, the course of AVB, feeding problems and respiratory monitoring.

### Outcomes

Primary outcome was food intake within the 24-h follow-up, reported by parents or nurses, and expressed as a percentage of the habitual intake. Habitual intake (i.e., not during the bronchiolitis episode) was reported by the parents. Bottle-feeding was reported in millilitres and other intakes in numbers of teaspoons that were converted to milligrams (1 spoonful = 5 mg).

The clinical condition was evaluated before the intervention and 5 min, 30 min and 24 h after the intervention using the respiratory score developed by Gajdos et al. [16]. This score involves the calculation and addition of scores for 3 domains: age-based respiratory rate (score of 1–3), retraction signs (score of 0–3) and wheezing (score of 0–3), with a total score ranging from 1 to 9. Higher scores indicate greater respiratory distress. HR and SpO2 were recorded within the same time frame on pulse oximeters (Philips IntelliVue MP5 ®, Philips Healthcare, Netherlands). Outcomes were collected with the child breathing room air, in a quiet environment. Children who were sleeping at the time of the evaluation were not woken up. Additionally, total sleep time and the number of vomit episodes were reported by parents or nurses on a form designed for the study. The mean SpO2 during the sleep time was extracted from the pulse oximeters; the mean saturation of each 5-min block was automatically calculated by the device and used for the analysis.

### Sample size calculation

Food intake as a percentage of habitual intake has never been reported as a clinical endpoint in similar studies. Therefore, the sample size calculation was based on the threshold feeding values used in clinical scores to determine the severity of bronchiolitis. Children with moderate bronchiolitis were expected to have a ~ 50% food intake at baseline, and a 25% difference during the 24 h of follow-up was hypothesized, corresponding to a major clinical change since a food intake > 75% is usually considered as adequate oral intake of fluids and feeds [5, 11]. As the heterogeneity of food intake improvement in 24 h is unknown in children with AVB, we expected a high variability and the standard deviation of the difference was set to 25%. Using a 90% power and a 5% alpha risk, a sample size of 42 children (21 per group) was required.

### Randomisation and blinding

Randomisation was performed with a computer-generated random number sequence (http://www.edgarweb.org.uk/). Allocation was concealed with sealed opaque envelopes until the child was enrolled. Before providing written informed consent, parents were informed that they would not attend the intervention and would be conducted to a waiting room outside the department to preserve blinding. The investigators in charge of the outcome measurements (MC, CM and GP) as well as the physicians and the nurses of the ward were blinded to group allocation.

### Statistical methods

Statistical analyses were performed by an independent professional statistician (RH). Continuous data were expressed as means (± SD) or medians (interquartile range) depending on the distribution, and categorical data were expressed as counts (%). Between-group differences for the primary outcome were modelled with an ordinary least squares linear regression for quantitative variables and expressed as the mean difference with corresponding 95% confidence intervals, adjusted to the child’s baseline food intake (at inclusion). The same method was applied to the secondary outcomes. Two-tailed p-values were calculated and considered as significant when < 0.05. An intention-to-treat analysis was performed for all the outcomes. All statistical analyses were performed with Stata version 17.1 (StataCorp LLC, College Station, Texas, USA).

### Compliance with the pre-registered protocol

Sleep quality assessment was planned as the amount of oxygen desaturation (SpO2 < 90%) during diurnal and nocturnal sleep. However, technical constraints led us to calculate mean SpO2 during sleep, on the basis of a continuous monitoring with the mean calculated every 5 min. There was no other departure from the planned study methods.

## Results

A total of 1118 children were screened for eligibility; 1061 did not met the inclusion criteria and main cause was breastfeeding and 12 declined to participate. Forty-two children were finally randomized and 40 completed the study (Fig. 1). Two children, one in each group, were excluded from the final analysis because they required enteral feeding within the 24-h follow-up. The data for the primary and secondary outcomes were available for all the 40 children that completed the study. The children were mainly boys (68%) with a mean age of 5.0 (± 2.9) months. The characteristics of the infants are described in Table 1, and including the 2 excluded children in the additional file 1. Full participants data are described in the additional file 2.

**Table 1:** Baseline characteristics of the participants

	**Total** **(*N* = 40)**	**PSET** **(*N* = 20)**	**Control** **(*N* = 20)**	**Mean or proportion** **difference with** **95%CI**	***P*-value**
Sex (male), n (%)	27 (68)	14 (70)	13 (65)	0.05 (-0.24 to 0.34)	1
Age (months), mean (± SD)	5.0 (± 2.9)	5.1 (± 3.1)	4.8 (± 2.8)	0.2 (-1.6 to 2.1)	0.80
Gestation (wk), mean (± SD)	38.8 (± 1.7)	38.3 (± 1.6)	39.3 (± 1.4)	-1.1 (-2.5 to 0.2)	0.10
Height (cm), mean (± SD)	62.4 (± 7.2)	61.7 (± 8.2)	63.0 (± 6.1)	-1.3 (-6.0 to 3.4)	0.57
Weight (kg), mean (± SD)	6.8 (± 1.8)	6.7 (± 1.8)	7.0 (± 1.8)	-0.3 (-1.4 to 0.9)	0.66
Eczema or history of atopy^a^, n (%)	21 (53)	13 (65)	8 (40)	0.25 (-0.05 to 0.56)	0.21
Prior bronchiolitis, n (%)	13 (33)	6 (30)	7 (35)	0.05 (-0.24 to 0.34)	1
Prematurity (35-37GA), n (%)	4 (10)	4 (20)	0 (0)	0.15 (-0.15 to 0.35)	0.11
Tobacco smoke exposure, n (%)	22 (55)	10 (50)	12 (60)	0.10 (-0.21 to 0.41)	0.75
Viral profile:					
RSV, n (%)	22 (55)	9 (45)	13 (65)	0.20 (-0.11 to 0.51)	0.35
Influenza, n (%)	5 (13)	3 (15)	2 (10)	0.05 (-0.25 to 0.35)	0.74
Rotavirus, n (%)	1 (3)	0 (0)	1 (5)	0.10 (-0.35 to 0.45)	0.51
Other, n (%)	12 (30)	8 (40)	4 (20)	0.20 (-0.05 to 0.40)	0.31
Duration of symptoms at randomisation (days), mean (± SD)	4.3 (± 2.0)	4.5 (± 2.6)	4.1 (± 1.1)	0.4 (-0.91 to 1.7)	0.53
Time interval admission to randomisation (hours), mean (± SD)	13.8 (± 5.7)	15.1 (± 5.9)	12.5 (± 5.2)	2.6 (-1.0 to 6.2)	0.15
Treatments:					
Bronchodilators, n (%)	18 (45)	10 (50)	8 (40)	0.10 (-0.21 to 0.41)	0.75
ICS, n (%)	8 (20)	4 (20)	4 (20)	0.0 (-0.28 to 0.28)	1
Antibiotics, n (%)	3 (8)	0 (0)	3 (15)	0.05 (-0.35 to 0.25)	0.23
Oxygen in the ED, n (%)	15 (38)	5 (25)	10 (50)	0.20 (-0.10 to 0.50)	0.19
Severity at admission:					
Respiratory rate (cpm), mean (± SD)	53.3 (± 12.2)	55.7 (± 11.5)	51.1 (± 12.7)	4.6 (-3.4 to 12.6)	*0.25*
Heart rate (bpm), mean (± SD)	156.5 (± 14.4)	154.8 (± 14.6)	158.3 (± 14.4)	-3.5 (-12.7 to 5.8)	*0.46*
SpO2 (%), mean (± SD)	96.7 (± 2.5)	97.1 (± 2.3)	96.4 (± 2.6)	0.8 (-0.8 to 2.3)	*0.34*
Temperature (°C), mean (± SD)	37.3 (± 0.6)	37.4 (± 0.6)	37.3 (± 0.6)	0.1 (-0.4 to 0.5)	*0.74*

Data are expressed as mean (SD) and as n (%)

*CI *Confidence interval, *ED* Emergency department, *GA* Gestational age, *ICS* Inhaled corticosteroids, *PSET *Prolonged slow expiration technique, *RSV* Respiratory syncytial virus, *SD* Standard deviation, *SpO2* Pulsed oxygen saturation, *wk *week

^a^History of atopy was defined as eczema or asthma in first-degree relatives

### Primary outcome measure

**Fig. 2:**
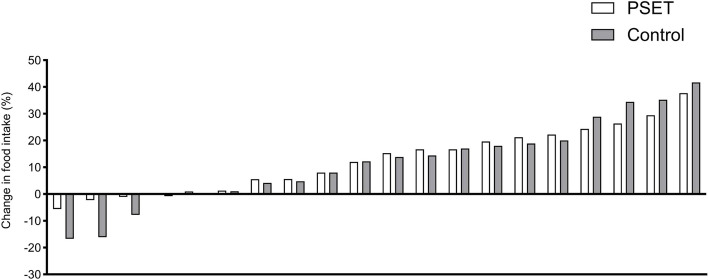
Individual percentage change in food intake during the 24 h of follow-up. White bars represent the PSET group and black bars represent the control group

Food intake was not different between groups at baseline (estimate: 2.6%, 95%CI: -9.3 to 14.3; *p* = 0.66), and increased during the 24 h of follow-up for most of the children (Fig. 2). PSET had no effect on food intake compared to standard treatment (estimate: 1.8%, 95%CI: -7.0 to 10.6; *p* = 0.68) (Table 2).

**Table 2:** Comparisons between PSET and control group

	**PSET** **(*N* = 20)**	**Control** **(*N* = 20)**	**Estimate (95%CI)**	***P*-value**
**Primary outcome:** Food intake (%), mean (± SD)				
- Baseline	59.3 (± 18.2)	56.7 (± 18.7)	2.6 (-9.3 to 14.3)	0.66
- 24 h following the intervention	72.0 (± 17.8)	68.3 (± 20.7)	1.8 (-7.0 to 10.6)	0.68
**Secondary outcomes:** Bronchiolitis Severity score (n), mean (± SD)				
- Before intervention	4.4 (± 2.2)	4.0 (± 2.2)	0.4 (-1.0 to 1.8)	0.56
- 5’	3.9 (± 2.2)	3.3 (± 2.1)	0.6 (-0.8 to 2.0)	0.39
- 30’	2.9 (± 1.7)	3.1 (± 1.7)	-0.2 (-1.3 to 0.9)	0.71
- 24 h	3.1 (± 1.9)	3.3 (± 1.9)	-0.2 (-1.5 to 1.1)	0.73
SpO2 (%), mean (± SD)				
- Before intervention	96.6 (± 2.6)	98.2 (± 1.4)	-1.6 (-3.0 to -0.3)	0.02
- 5’	97.8 (± 1.9)	97.9 (± 2.3)	-0.1 (-1.5 to 1.2)	0.83
- 30’	96.6 (± 3.0)	97.3 (± 2.4)	-0.7 (-2.4 to 1.0)	0.42
- 24 h	97.0 (± 3.0)	98.6 (± 1.4)	-1.6 (-3.2 to 0.1)	0.06
RR (cpm), mean (± SD)				
- Before intervention	50.0 (± 12.9)	47.1 (± 9.2)	2.9 (-4.3 to 10.1)	0.43
- 5’	49.1 (± 10.5)	47.7 (± 10.2)	1.4 (-5.2 to 8.0)	0.67
- 30’	42.2 (± 9.1)	42.8 (± 8.8)	-0.6 (-6.3 to 5.1)	0.83
- 24 h	48.3 (± 10.9)	46.1 (± 10.0)	2.2 (-4.8 to 9.3)	0.53
HR (cpm), mean (± SD)				
- Before intervention	150.5 (± 14.2)	153.3 (± 12.9)	-2.8 (-11.5 to 5.9)	0.52
- 5’	145.6 (± 13.8)	158.4 (± 10.8)	-12.8 (-20.7 to -4.9)	< 0.01
- 30’	137.6 (± 14.5)	136.3 (± 18.2)	1.4 (-9.2 to 11.9)	0.80
- 24 h	149.3 (± 14.9)	148.3 (± 15.6)	0.9 (-9.4 to 11.3)	0.85
Number of vomit episodes within 24 h, mean (± SD)	0.9 (± 1.2)	0.5 (± 0.7)	0.4 (-0.2 to 1)	0.21
Total sleep time within 24 h, mean (± SD)	13.5 (± 1.7)	14.6 (± 2.4)	-1.1 (-2.5 to 0.3)	0.11
SpO2 during sleep, mean (± SD)	96.9 (± 1.8)	97.2 (± 1.0)	-0.3 (-1.2 to 0.7)	0.58

Data are expressed as mean (± SD)

*CI* Confidence interval, *HR* Heart rate, *PSET* Prolonged slow expiration technique, *RR* Respiratory rate, *SD* Standard deviation, *SpO2* Pulsed oxygen saturation

### Secondary outcome measures

A marginal difference in the baseline SpO2 was observed, with a lower SpO2 in the PSET group (estimate -1.6%, 95%CI: -3.0 to -0.3; *p* = 0.02). No differences were observed on SpO2 in the other endpoints. No difference was observed in clinical severity and RR over the different endpoints. Baseline HR was similar between groups and a lower HR at 5 min was observed in the PSET group (estimate -12.8 bpm, 95%CI: -20.7 to -4.9; *p* < 0.01), which was not confirmed in the following measurements (Table 2). Additionally, PSET had no effect on the number of vomit episodes (estimate: 0.4, 95%CI: -0.2 to 1; *p* = 0.21), nor on the total sleep time (estimate: -1.1 h, 95%CI: -2.5 to 0.3; *p* = 0.11) and mean SpO2 during sleep (estimate: -0.3%, 95%CI: -1.2 to 0.7; *p* = 0.58).

## Discussion

Our findings indicate that PSET had no effect on the 24-h food intake of children hospitalized with moderate AVB. Also, PSET did not modified the clinical condition on the respiratory score, nor the RR, HR and SpO2 at any of the follow-up assessments. We also observed that PSET did not modified the total sleep duration or the SpO2 during sleep, and had no impact on the number of vomits in the 24 h following the intervention.

PSET did not show any benefit on our endpoints in the present study. Yet, airway clearance is a symptomatic reference treatment for children with chronic respiratory diseases when the main symptom is bronchial congestion (e.g., cystic fibrosis or neuromuscular disease) [20]. The effectiveness of ACT in improving gas exchange and decreasing respiratory symptoms has been demonstrated in these groups, at least in the short term [21, 22]. However, when it comes to AVB, it appears that the obstruction is not solely characterized by bronchial congestion. Multifactorial bronchial obstruction could be related to other mechanisms such as inflammation, oedema and bronchospasm [2]. Hence, it is unlikely that ACT (for instance PSET) can have a substantial impact on symptoms, as we observed in the present study. This raises another problem, which is the identification of children with AVB potentially responsive to ACT (i.e., those with a bronchial obstruction dominated by mucus plugging). Our study proposed an original model by including infants likely to benefit from the PSET intervention after a double assessment by a physician and an experienced physiotherapist. However, since we did not find any change in food intake, this procedure could have failed to identify such children. Indeed, the tools that we used: stethoscope examination, subjective cough assessment and respiratory sounds are neither reliable nor objective, but no other tool currently meets these criteria [23, 24]. The other explanation that cannot be excluded is that the relief of symptoms with ACT described in previous studies is insufficient to induce changes on a clinically relevant outcome like food intake [9].

Contrary to the latest Cochrane meta-analysis findings [9], in the present study PSET did not transiently improved the clinical severity of bronchiolitis. This difference could be explained by the use of many different respiratory tools with heterogenous clinimetric properties in the Cochrane meta-analysis. In our study, we chose to use a tool that was previously identified as one of the best available based on clinimetric performance [25]. Another key point that we noticed during the study is that many of the children were sleeping at the time of the assessments, especially the 30-min assessment. However, studies showing that ACT could be beneficial do not always specify the child’s state of arousal, although it would have a crucial impact on respiratory scores (i.e., lower HR and RR, fewer retractions and respiratory sounds) [12, 14]. Furthermore, children receiving ACT could experience respiratory destabilization and fatigue, reinforcing the probability of sleep at the time of the assessments 30 min later. Moreover, in routine clinical practice, it is recommended to assess the infant’s clinical status when awake and calm [5]. This was not anticipated in the present study, and we were unable transcribe which children were sleeping at the time of the assessment accurately. We strongly encourage anticipation and clarification of this issue in future studies.

Food intake and clinical severity improved for most of the children during the 24-h follow-up. This is likely because of the natural positive evolution of AVB, as well as the application of upper airway clearance involving a nasal instillation. Nasal instillation, with or without RRC, is known to improve oxygenation and reduce respiratory distress, at least in the short term [18, 26]. It is recommended in AVB to maintain upper airway patency, regardless of the technique used [2, 5]. However, upper airway clearance and ACT are often confused or mixed up in studies evaluating ACT in AVB [13, 14]. This sometimes leads to the conclusion that ACT is effective although the effect of upper airway clearance itself has not been controlled for. In our study, all the children received the same upper airway clearance protocol during the 24-h follow-up, which allows us to draw conclusions on the specific impact of the PSET.

### Strengths and limitations

To our knowledge, one important novelty of the present study is that we measured the effect of PSET on food intake for the first time. Food intake is a highly prevalent symptom in AVB, an objective reflection of the clinical condition of infants over the course of the disease, and also an important source of parental anxiety. In line with the conclusions of the latest guidelines, the present study was randomized and controlled, and assessors, parents and all practitioners caring for the infants were blinded to group allocation. Finally, an important finding for the future is that this study included hospitalized children with moderate AVB, which is similar to the profile of children seen in outpatient settings. The literature is scarce regarding the management of children with AVB in ambulatory settings and this study could be a starting point for other work on the subject.

This study has limitations. Breastfed children were not included. The methods currently available to quantify human milk intake (i.e., test-weighing, direct observation or isotope dilution techniques) have been judged to be either too costly, ethically difficult to implement or too imprecise [27–29]. The external validity of our results is therefore limited. In addition, feeding in hospital is necessarily disrupted compared to home. Parents may not have all the equipment in the room and children may be disturbed but this issue affected both groups equally and probably did not influence our results. Finally, the difference in food ingestion expected in the sample size calculation (+ 25%) was high. In the absence of literature, we based our sample size calculation on a difference that would have made a major clinical change according to clinical severity scales, but which was not found in this study. Hence, the wide confidence interval of the 24-h food ingestion difference (-7.0% to + 10.6%) would have been more precise with a larger sample.

The respiratory score at the initial assessment was sometimes < 4 or > 6 points as there could be a 30-min delay between the inclusion visit and the initial assessment. This again highlights how these scores fluctuate and reinforces the idea of being cautious when judging the effect of an intervention on the basis of these scores.

## Conclusions

PSET had no effect on the 24-h food intake compared with standard treatments including upper airway clearance in hospitalized children with moderate AVB.

## Supplementary Information

Supplementary Material 1.

Supplementary Material 2.

Supplementary Material 3.

## Data Availability

All data generated or analyzed during this study are included in this published article and its supplementary information files.

## Abbreviations

AAD: Assisted autogenic drainage
AVB: Acute viral bronchiolitis
ACT: Airway clearance techniques
RR: Respiratory rate
HR: Heart rate
SpO2: Pulsed oxygen saturation
PSET: Prolonged slow expiration technique
RRC: Retrograde rhinopharyngeal clearance
