# Supplementary material for: Effect of a prolonged slow expiration technique on 24-h food intake in children hospitalized for moderate bronchiolitis: a randomized controlled trial
Source: Ital J Pediatr. 2024 Sep 27;50:196. doi: 10.1186/s13052-024-01770-2 (PMC11438082; doi:10.1186/s13052-024-01770-2)
Supplement: Supplementary file 1 — Supplementary Material 1. [file 13052_2024_1770_MOESM1_ESM.docx]

**S1 Table. Baseline characteristics of the whole sample of participants including the two children subsequently excluded**

|  | **Total**  **(N=42)** | **PSET**  **(n=21)** | **Control**  **(n=21)** | **Mean or proportion**  **difference with**  **95%CI** | **P-value** |
| --- | --- | --- | --- | --- | --- |
| Sex (male), n (%) | 28/14 | 14/7 | 14/7 | 0.0 (-0.29 to 0.29) | 1 |
| Age (months), mean (±SD) | 4.8 (±2.9) | 4.9 (±3.1) | 4.7 (±2.8) | 0.2 (-1.6 to 2.0) | 0.81 |
| Gestation (wk), mean (±SD) | 38.8 (±1.6) | 38.3 (±1.8) | 39.3 (±1.3) | -1.0 (-2.4 to 0.3) | 0.08 |
| Height (cm), mean (±SD) | 62.1 (±7.1) | 61.3 (±8.2) | 62.8 (±6) | -1.5 (-6.0 to 3.0) | 0.50 |
| Weight (kg), mean (±SD) | 6.7 (±1.8) | 6.6 (±1.8) | 6.9 (±1.8) | -0.3 (-1.4 to 0.9) | 0.65 |
| Eczema or history of atopy*, n (%) | 22 (52) | 14 (67) | 8 (38) | 0.29 (-0.02 to 0.59) | 0.12 |
| Prior bronchiolitis, n (%) | 13 (31) | 6 (29) | 7 (33) | 0.05 (-0.20 to 0.30) | 1 |
| Prematurity (35-37GA), n (%) | 4 (10) | 4 (19) | 0 (0) | 0.15 (-0.15 to 0.35) | 0.11 |
| Tobacco smoke exposure, n (%) | 22 (52) | 10 (48) | 12 (57) | 0.1 (-0.21 to 0.40) | 0.76 |
| Viral profile:  RSV, n (%)  Influenza, n (%)  Rotavirus, n (%)  Other, n (%) | 24 (57)  5 (12)  1 (2)  12 (29) | 10 (48)  3 (14)  0 (0)  8 (38) | 14 (67)  2 (10)  1 (5)  4 (19) | 0.19 (-0.11 to 0.49)  0.05 (-0.25 to 0.35)  0.10 (-0.35 to 0.45)  0.16 (-0.10 to 0.42) | 0.36  0.85  0.70  0.40 |
| Duration of symptoms at randomisation (days), mean (±SD) | 4.3 (±1.9) | 4.5 (±2.6) | 4.1 (±1) | 0.42 (-0.79 to 1.65) | 0.48 |
| Time interval from admission to randomisation (hours), mean (±SD) | 13.7 (±5.9) | 15.3 (±5.9) | 12 (±5.5) | 3.3 (-0.26 to 6.9) | 0.07 |
| Treatments:  Bronchodilators, n (%)  ICS, n (%)  Antibiotics, n (%)  Oxygen in the ED, n (%) | 18 (43)  9 (21)  4 (9)  15 (36) | 10 (48)  5 (24)  0 (0)  5 (24) | 8 (38)  4 (19)  4 (19)  10 (48) | 0.10 (-0.21 to 0.41)  0.05 (-0.25 to 0.30)  0.0 (-0.30 to 0.30)  0.20 (-0.10 to 0.50) | 0.76  1  0.11  0.20 |
| Severity at admission:  Respiratory rate (cpm), mean (±SD)  Heart rate (bpm), mean (±SD)  SpO2 (%), mean (±SD)  Temperature (°C), mean (±SD) | 53 (±12)  156.1 (±14.4)  96.7 (±2.5)  37.3 (±0.7) | 55 (±11.7)  154.9 (±14.3)  97.2 (±2.4)  37.3 (±0.7) | 51.1 (±12.4)  157.3 (±14.7)  96.2 (±2.6)  37.3 (±0.7) | 3.8 (-3.9 to 11.5)  -2.4 (-11.5 to 6.6)  1.0 (-0.5 to 2.6)  0.0 (-0.46 to 0.39) | 0.32  0.59  0.20  0.87 |

*Data are expressed as mean (SD) and as n (%). * History of atopy was defined as eczema or asthma in first-degree relatives*

*CI: confidence interval; ED: emergency department; GA: gestational age; ICS: inhaled corticosteroids; PSET: prolonged slow expiration technique; RSV: respiratory syncytial virus; SpO2: pulsed oxygen saturation; wk: week*
